# Exploiting structural and topological information to improve prediction of RNA-protein binding sites

**DOI:** 10.1186/1471-2105-10-341

**Published:** 2009-10-18

**Authors:** Stefan R Maetschke, Zheng Yuan

**Affiliations:** 1 Institute for Molecular Bioscience, The University of Queensland, QLD 4072, Australia; 2 Institute for Molecular Bioscience and ARC Centre for Excellence in Bioinformatics, The University of Queensland, QLD 4072, Australia

## Abstract

**Background:**

RNA-protein interactions are important for a wide range of biological processes. Current computational methods to predict interacting residues in RNA-protein interfaces predominately rely on sequence data. It is, however, known that interface residue propensity is closely correlated with structural properties. In this paper we systematically study information obtained from sequences and structures and compare their contributions in this prediction problem. Particularly, different geometrical and network topological properties of protein structures are evaluated to improve interface residue prediction accuracy.

**Results:**

We have quantified the impact of structural information on the prediction accuracy in comparison to the purely sequence based approach using two machine learning techniques: Naïve Bayes classifiers and Support Vector Machines. The highest AUC of 0.83 was achieved by a Support Vector Machine, exploiting PSI-BLAST profile, accessible surface area, betweenness-centrality and retention coefficient as input features. Taking into account that our results are based on a larger non-redundant data set, the prediction accuracy is considerably higher than reported in previous, comparable studies. A protein-RNA interface predictor (PRIP) and the data set have been made available at .

**Conclusion:**

Graph-theoretic properties of residue contact maps derived from protein structures such as betweenness-centrality can supplement sequence or structure features to improve the prediction accuracy for binding residues in RNA-protein interactions. While Support Vector Machines perform better on this task, Naïve Bayes classifiers also have been found to achieve good prediction accuracies but require much less training time and are an attractive choice for large scale predictions.

## Background

RNA-protein interactions are pivotal for many fundamental cellular functions such as transcriptional regulation, splicing and protein synthesis. Thus the identification of RNA binding sites is essential for the understanding of a variety of biological processes. In general, computational methods to predict interface residues for an individual protein fall into two major categories: sequence-based and structure-based. Most published studies have extensively used the information derived from protein sequence.

One of the earliest attempts to predict binding residues in RNA-protein interfaces was performed by Jeong et al. [1]. They utilized a neural network with amino acid type and secondary structure information as input features. The method achieved a Matthews correlation coefficient (MCC) of 0.29 for 10-fold cross-validation on a data set with 96 chains from 58 protein-complexes. A post processing step (state shifting and filtering) improved the accuracy further but required information usually not available in the query phase [2].

Furthermore, Jeong et al. [3] studied different methods to calculate profiles and improved their previous results [1] by utilizing weighted PSI-BLAST profiles to a MCC of 0.41. However, they used a data set containing 86 proteins with sequence similarities up to 70% and the accuracy was not calculated via strict cross-validation tests.

Wang et al. [4] applied support vector machines (SVMs) with RBF kernels and artificial neural networks (ANNs) to predict DNA and RNA binding residues. Sequence features such as side chain *pK*_*a *_value, the Kyte-Dolittle hydrophobicity scale and molecular mass were exploited. They reported a specificity of 69.9% and a sensitivity of 66.3% with five-fold cross-validation on residue-level. By including additional features such as accessible surface area and conservation score [5], they improved their previous results. Using SVMs, an AUC of 0.75 (65.8% sensitivity, 75.7% specificity) on a data set of 107 non-redundant protein chains was achieved. Down-sampling was applied to balance positive and negative samples of the data set, which resulted in better performance in comparison to the unbalanced case.

Kim et al. [6] studied the propensities of individual amino acids and amino acid pairs in RNA-protein interfaces. They reported 50% sensitivity and 57% specificity for a method that combined averaged singlet and doublet propensities.

A recent predictor by Terribilini et al. [2,7] utilized a Naive Bayes classifier to predict the residues involved in RNA-protein interaction based on amino acid propensities. On a larger data set, with lower sequence similarity than Jeong's [1], a correlation coefficient of 0.35 was achieved (specificity: 51%, sensitivity: 38%). Surprisingly, additional information such as secondary structure, relative accessible surface area, sequence entropy, hydrophobicity or electrostatic potential was not found to improve the prediction accuracy. In a comparison of Terribilini's and Jeong's methods, both predictors achieved very similar accuracies on Jeong's data set.

Kumar et al. [8], using a SVM with a second order polynomial kernel and PSI-BLAST [9] profiles as input features, achieved an MCC of 0.45 (specificity: 89.6%, sensitivity: 53.0%) on Jeong's data set [1] (86 protein chains). On a larger, more recent data set (107 protein chains) with lower sequence similarity (25%) by Wang et al. [4], a significantly lower MCC of 0.32 was reached due to the overestimation on a redundant data set.

The focus of a recent paper by Shazman et al. [10] was on the differentiation of non-binding and RNA-binding proteins based on electrostatic properties - not on the prediction of binding residues per se. However, they also measured the overlap between positively charged surface patches and the actual binding sites and found dramatic variations ranging from 0% to 100%, indicating that positive charge alone is a comparitvely weak predictor for binding residues.

A very high prediction accuracy, with a MCC of 0.50, has been reported very recently by Spriggs et al. [11] on a data set comprised of 81 RNA-binding proteins (RNAset81), derived from Kumar's data set [8]. It is however to note that this data set is small and only weakly redundancy reduced (up to 70% sequence similarity). A SVM with an RBF kernel was utilized to analyze input features such as sequence profiles, interface propensities, accessibility and hydrophobicity. On an independent test set the predictor achieved a MCC of 0.41.

With the constantly increasing number of known 3D structures of RNA binding proteins, it is possible to use more and more structural features to leverage accurate prediction. Recently, Chen and Lim [12] investigated physicochemical and geometrical properties, together with conservation score obtained from sequence alignments, to predict RNA-binding sites. However, it is difficult to compare this approach with previous methods based on prediction performance.

In this study, we systematically study sequential, graph-topological and spatial features with respect to their predictive power for the identification of residues involved in RNA-protein interaction. We have implemented two methods based on Naïve Bayes classifiers and Support Vector Machines, using residue PSI-BLAST profiles and sequential neighbors as input to predict RNA binding sites. The accuracy of these classifiers serves as a baseline that reflects the performance of sequence-based methods.

Secondly, we study different graph-theoretic properties that may be associated with interface residues, where protein structures are represented as graphs derived from residue contacts. Features such as closeness centrality and betweenness centrality were found to be useful in predicting enzyme active sites and ligand-binding sites [13], identifying critical residues for protein function [14] and analyzing protein-protein interactions [15,16]. However, it is not known yet what types of graph-theoretic features are correlated with protein-RNA interaction and therefore contributing to the prediction.

By carefully examining seven topological features, we found betweenness centrality to be the most predictive feature, which can be used to enhance prediction accuracy. Instead of using sequential neighbors to encode the input feature vector as in sequence-based methods, we utilize structural information by taking into account network topological or spatial neighbors to improve the prediction performance. The prediction accuracy of our method has been evaluated on two large, non-redundant data sets and a peak AUC of 0.83 was reached (five-fold cross-validation). We furthermore created a new independent test set (RB36), where our method achieved an AUC of 0.77.

## Results and Discussion

We have investigated sequential, graph-theoretic and spatial features that are predictive for binding residues in RNA-protein interfaces. In particular, we were interested in estimating the impact of structural information on the prediction accuracy in comparison to a purely sequence based approach.

### Predictive power of amino acid indices

As a first step, we measured the predictive power for binding residues of all amino acid indices, available in the AAIndex database [17]. For each residue in a protein chain the corresponding value within an AAIndex scale was selected. The predictive power of a scale was then calculated as the *Area under the ROC curve *(AUC) [18] over all residues within the RB144 data set, which contains 144 Protein-RNA complexes with annotated binding residues. Note that no classifier and therefore no cross-validation scheme is required to compute the AUC estimates at this stage. The ten scales with the highest AUCs are listed in Table 1. Residues involved in RNA-protein interfaces are known to show a preference for hydrophobic amino acids [2,6], which is reflected by the results in Table 1. COWR900101, JURD980101 and ROSM880102 are essentially hydrophobicity scales. Similarly, scales that discriminate between inside and outside residues (RADA880107, CHOC760103, OLSK800101) and scales related to the partition coefficient (GUYH850105, GARJ730101), which is a measure for lipophilicity [19], are most predictive for interface residues.

**Table 1:** Predictive power of amino acid indices.

**AUC**	**ID**	**Description**
0.646	GUOD860101	Retention coefficient at pH 2
0.644	GUYH850105	Apparent partition energies calculated from Chothia index
0.643	RADA880107	Energy transfer from out to in
0.643	CHOC760103	Proportion of residues 95% buried
0.642	OLSK800101	Average internal preferences
0.640	COWR900101	Hydrophobicity index, 3.0 pH
0.639	JURD980101	Modified Kyte-Doolittle hydrophobicity scale
0.639	ROSM880102	Side chain hydropathy, corrected for solvation
0.638	TANS770106	Normalized frequency of chain reversal D
0.637	GARJ730101	Partition coefficient

Table of the ten amino acid indices with the highest predictive power (AUC) on the RB144 data set.

Scale TANS770106 is derived from a one-dimensional short-range interaction model for specific sequence copolymers of amino acids and is related to protein conformation [20]. It may appear as a high ranking scale due to a bias of the sample set toward aminoacyl-tRNA synthetases, many of which are allosteric in nature.

The highest ranking scale GUOD860101 [21] describes the retention coefficient (a coefficient related to the partition coefficient) for Peptide Nucleic Acids (PNAs), which are synthetic biopolymers chemically similar to DNA and RNA.

**Figure 1:**
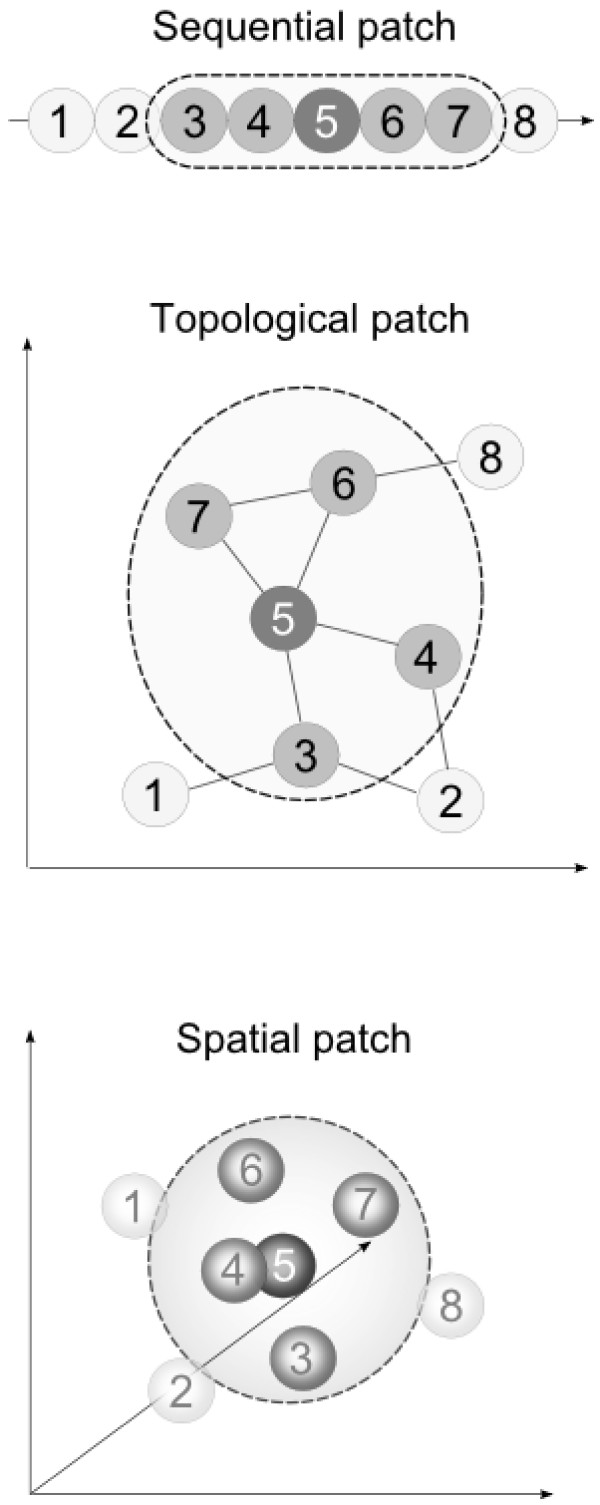
**Visualization of patch types**. Cartoon of a sequential (top), topological (center) and spatial (bottom) patch of size five.

Although Table 1 does not reveal novel characteristics of interface residues, it establishes a base line for the prediction accuracy of classifiers based on single residue features. Previous work has shown that taking the neighborhood of an interface residue into account significantly improves the accuracy for classifying a residue as interacting or non-interacting [2]. Consequently we studied three different types of neighborhood patches (sequential, topological and spatial, see Figure 1) to incorporate neighboring residues and evaluated the prediction performance in dependence of the patch size and patch type.

### Predictive power of residue patches

**Figure 2:**
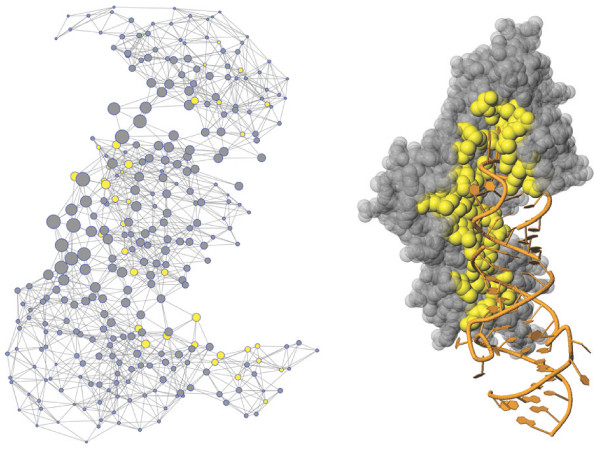
**Contact graph and tertiary structure of 1R3E:A**. Contact graph and tertiary structure of 1R3E:A. Binding residues are marked in yellow within the graph and the structure. RNA is displayed as cartoon in orange. Graph layout according to the Kamada-Kawai algorithm [33] and generated by the JUNG library. Node size proportional to averaged betweenness centrality (spatial patch with size 19). Note that edge lengths and node positions are not related to the spatial location of residues in the 3D structure.

*Sequential patches *(or sequence sliding windows) of size *n *for sequential data are constructed by extracting the *n *residues nearest (sequential distance) to the residue (center residue), which is to be classified. For topological and spatial features the definition of a patch of neighboring residues requires more consideration. We define a *spatial patch *of size *n *as the set of the *n *residues with the smallest euclidean distance between their *C*_*α*_-atoms and the *C*_*α*_-atom of the residue in the center of the patch. This approach was also used by Tjong and Zhou to predict protein-DNA binding sites [22]. A *topological patch *is similarly defined by the *n *vertices with the smallest geodesic distances (shortest paths) to the center vertex. The underlying graph is thereby derived from a map of residue contacts (see Material and Methods and Figure 2).

To construct a feature vector with ordered elements from a spatial or topological patch, the features associated with the residues or nodes of the patch were sorted according to distance. For a topological patch the geodesic distances, and for a spatial patch the euclidean distances to the patch center were employed. In the case of equal distances, the sequential distance within the primary sequence was used as an additional criterion.

To achieve optimal classification accuracy and to identify the typical size of the neighborhood that contributes to the binding propensity of an interface residue, we measured the prediction accuracy for the different patch types for patch sizes varying from 1 to 30 residues.

**Figure 3:**
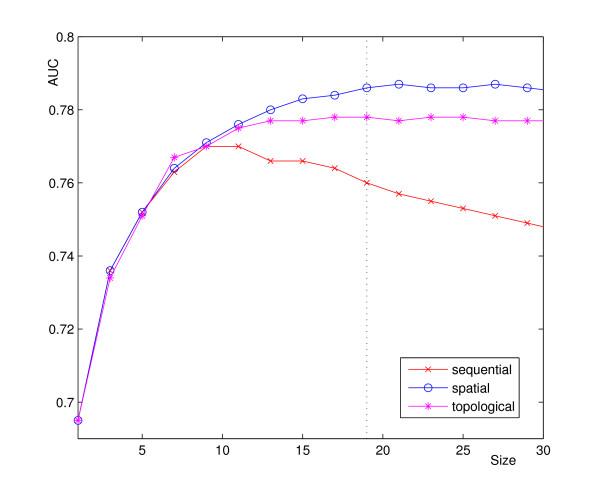
**Performance comparison patch types and sizes**. Prediction accuracy (AUC) on the RB144 data set for three patch types and varying patch sizes. Prediction by a Naive Bayes classifier with PSI-Blast profiles as residue features.

Figure 3 shows the five-fold cross-validation prediction accuracy (AUC) of a Naive Bayes classifier over increasing patch sizes for the three patch types on the RB144 data set. We chose a Naive Bayes classifier for this step of the study, since the method is fast, has no control parameters that require optimization, and has shown good performance for this classification problem [2,7]. Similarly, we chose profile information as input, which Jeong et al. [1] has exploited with good success. In all the three types of patches, each residue was encoded by its PSI-Blast profile, resulting in a feature vector with 21 times the patch size elements. The performance curve of the sequential patch in Figure 3 shows a peak AUC for a patch size of 11 residues and then declines quickly due to border effects and the inclusion of more and more spatially unrelated residues into the patch. While the size is critical for the sequential patch, the performance of the topological and the spatial patch is clearly less sensitive to larger patch sizes. Furthermore is the maximum AUC of the topological and the spatial patch higher than that of the sequential patch. Both reach a plateau for a patch size of roughly 19 residues, with the spatial patch achieving a top AUC of 0.79. Naive Bayes classifiers assume statistical independence of their input features. It is known however that there is a bias in the types of amino acids surrounding an interface residue [2]. Consequently, more advanced machine learning methods, with less strict independence assumptions, such as SVMs can be expected to achieve higher prediction accuracies. To validate this expectation, we trained SVMs with RBF-kernels for the three patch types, utilizing the optimal patch sizes determined above.

Table 2 compares the achieved prediction accuracies of the Naive Bayes classifiers and the SVMs with respect to patch type. All results are five-fold cross-validated on chain level for the RB144 data set. C-value (1.0) and *γ*-factor (0.01) for the SVM were optimized on a subset of the RB144 data set. Since the data set is heavily unbalanced, the cost factor (sample weights) for the classifiers was set to 5.7 in accordance to the proportion of binding and non-binding residues.

**Table 2:** Prediction performance for different patch types.

**Classifier**	**Patch type**	**AUC**	***δ***_**95**_	**MCC**	**SN[%]**	**SP[%]**
NB	sequential	0.77	0.016	0.30	73.6	66.8
NB	topological	0.78	0.016	0.31	75.4	65.7
NB	spatial	0.79	0.016	0.32	75.4	67.1

SVM	sequential	0.79	0.021	0.34	78.4	65.8
SVM	topological	0.80	0.021	0.34	79.9	64.4
SVM	spatial	0.80	0.021	0.36	80.0	65.6

Prediction performance for different patch types (sequential, topological, spatial) and classifiers (NB, SVM) on RB144 data set, tested by five-fold cross-validation. *C*-value for SVM was 1.0 and *γ*-value of RBF-kernel was 0.01. Cost factor was assigned as 5.7 to compensate for the unbalanced class distribution.

The results confirm that the SVMs significantly (*p *< 0.05) outperform the Naive Bayes approach. Furthermore, the spatial patch performed generally better than the topological patch, which performed better than the sequential patch. However, the differences in prediction accuracy were small, which can be explained by the fact that there is a considerable overlap of residues between the different patch types. For instance, in the case of the topological and spatial patch 80% of the patch residues overlap.

The highest AUC of 0.80 (Sensitivity 80%, Specificity 65%) was achieved by a SVM with a spatial patch. While the absolute improvements in AUC in relation to the Naive Bayes approach are small, the MCC is increased by approximately 10%. However, taking into account that the SVM is several orders of magnitude slower to train and test, the Naive Bayes approach is a valid alternative for large scale data analysis.

### Predictive power of graph-theoretical and geometrical features

Here, we aimed to identify features besides the profile that have high predictive power for interface residues, with the final goal to improve performance by combining highly predictive features. For this purpose we compared the prediction accuracy of the best amino acid propensity scale, the retention coefficient (RC) (see Table 1), with structural and topological features, such as accessible surface area (ASA) and betweenness centrality (BC).

The results presented in Figure 3 indicate a higher performance for features that consider the neighborhood of the residue to classify. In addition to feature values for individual residues we therefore also calculated averaged feature values over patches of residues. Note that in both cases only a single feature value for the residue of interest is computed. Consequently, the predictive power (AUC) of a feature could be calculated without involvement of a classifier and time-consuming cross-validation tests. Table 3 lists the prediction performance (AUC) of the features evaluated on the RB144 data set. Taking the peak values from Figure 3, we chose a patch size of 11 residues for sequential patches and a size of 19 for topological or spatial patches. Patch types and sizes are annotated in the related table columns. A patch size of one indicates the evaluation of a feature for the center residue only (no patch is used and no average is calculated).

**Table 3:** Predictive power of features on RB144 data set.

**AUC**	**Type**	**Size**	**Feature**
0.65	-	1	Retention coefficient (RC)
0.66	sequential	11	Averaged retention coefficient (saRC)
0.69	topological	19	Averaged retention coefficient (taRC)
**0.69**	spatial	19	Averaged retention coefficient (aRC)
**0.70**	-	1	Accessible surface area (ASA)
0.69	spatial	19	Averaged Accessible surface area (aASA)
0.69	-	1	Relative accessible surface area (rASA)
0.68	spatial	19	Averaged Relative accessible surface area (arASA)
0.66	spatial	19	Density (D)
0.56	-	1	Betweenness centrality (BC)
**0.71**	topological	19	Averaged betweenness centrality (aBC)
0.69	-	1	Status (S)
0.69	topological	19	Averaged Status (aS)
0.62	-	1	Cluster coefficient (CC)
0.64	topological	19	Averaged cluster coefficient (aCC)
0.64	-	1	Degree (G)
0.64	topological	19	Averaged degree (aG)
0.63	-	1	Eccentricity (E)
0.64	topological	19	Averaged eccentricity (aE)
0.62	-	1	Closeness (C)
0.64	topological	19	Averaged closeness (aC)

Predictive power (AUC) of features on RB144 data set. Patch type and patch size are listed in columns two and three. A missing patch type and patch size of one indicate features evaluated for individual residues (no patch used).

While the optimal patch sizes identified in Figure 3 are likely to be a reasonable choice, they are not necessarily optimal for features other than PSI-Blast profiles. However, to allow for a stringent comparison of different features, we limited our study to these two patch sizes and did not optimize the patch sizes individually for all the features explored.

Note that topological features, such as betweenness centrality (BC) for instance, are calculated based on the entire contact map of a protein chain. If a patch is used, the feature value for the center residue is computed as the average over all BC values of the patch residues. A detailed description of the evaluated features is provided in the Material and Methods section.

The results in Table 3 show that features averaged over patches generally achieve AUCs higher than or comparable to features for individual residues (patch size one). The only exceptions are the accessible surface area (ASA) and the relative accessible surface area (rASA), which both show slightly better performance for individual residues.

We also compared the performance of averaged retention coefficients (saRC, taRC, aRC) for the different patch types and the results show that spatial and topological patches are superior (AUC = 0.69) to the same feature calculated over the sequential patch (AUC = 0.66).

The solvent accessible surface area (ASA) measures whether a residue is located on protein surface and has been proven to be highly correlated with interface residues [23]. We have examined four different versions of accessible surface area: ASA and rASA for individual residues (patch size equals one) or averaged over a spatial patch (arASA, aASA). We found that the utility of the absolute ASA for individual residues yields the best result (AUC = 0.70).

**Figure 4:**
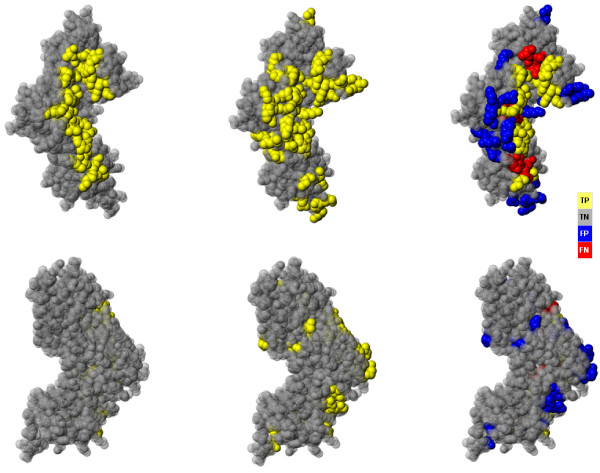
**Binding residue prediction for 1R3E:A**. Top row shows the front and bottom row shows the back of 1R3E:A (tRNA pseudouridine synthase. Left column: Protein structure and true binding site (yellow). Center column: Predicted binding site (yellow) by the Support Vector Machine (all features). Right column: Residue classification of 1R3E:A by the Support Vector Machine (all features), MCC 0.51, AUC 0.81, SN 71%, SP 90%. True positives are in yellow, true negatives are in gray, false positives are in blue and false negatives are in red. Diagrams are genereated with JMol .

Table 3 compares a number of graph theoretic properties. The topological feature with the highest predictive power was the averaged betweenness centrality (aBC) with an AUC of 0.71. Betweenness centrality reflects how heavily a residue is involved in the communication of residues (shortest paths), demonstrating its central role in the network. Interestingly the predictive power of betweenness centrality is very low for individual residues (BC) but is highly predictive when averaged over a patch of neighboring residues. This may suggest that a number of residues with higher betweenness centralities form a community to play a significant role in protein-RNA interaction. Figure 4 shows the contact graph of tRNA Pseudouridine Synthase (PDB ID: 1R3E). Previous work [15] suggested that betweenness centrality is associated with *hot spot *residues in protein-protein interfaces. Similarly, our study strongly suggests that this feature may also reflect the organization of residues located at protein-RNA interfaces.

Because a sole feature cannot accurately predict interface residues, combining features with high predictive power is a standard method to improve the overall accuracy. However, such a combination is only successful if the features to combine are not redundant. All graph-theoretic features in Table 3 are essentially centrality measures, which are typically highly correlated. We therefore picked only averaged betweenness centrality (aBC) and calculated the correlation coefficients between aBC and the two other top ranking features, such as ASA and aRC. The highest correlation coefficient of 0.33 was identified between ASA and aRC. ASA and aBC showed the lowest correlation (0.04), and the correlation coefficient for aBC and aRC was 0.17. The correlation between the three features was regarded as sufficiently low to justify their combination.

### Combination of highly predictive features

We studied the predictive power of features by averaging over patches of residues, which may not fully reflect their power, but is an effective way for feature selection. To gain an increase in prediction accuracy, we used machine learning methods such as Naive Bayes classifiers and Support Vector Machines to combine the feature values of the residues within a patch.

This is achieved by encoding a patch of residues as a feature vector, where each residue within the patch is represented by the corresponding feature value or values. For instance, a patch of size 11 with PSI-BLAST profile and retention coefficient as features is encoded as a vector containing 11 × (21 + 1) = 242 elements. As described in Section Methods, the residues (and consequently the features within the vector) are sorted according to their distance to the center residue.

We have observed changes in prediction accuracy when including more and more information, starting with information that can be derived from the primary sequence only, over topological information, up to structural information. To this purpose we assessed the prediction accuracy of different combinations of the PSI-BLAST profile feature with the three best performing features (ASA, aRC, aBC), identified in the previous section. Table 4 shows the results of this comparison, using a Naive Bayes classifier (NB) and Support Vector Machine (SVM) with an RBF-Kernel (*C*-value = 1.0, *γ*-value = 0.01, cost factor = 5.7). From Table 4 three trends become obvious. Firstly, as expected, the more information is included the higher is the prediction accuracy. Secondly, the Support Vector Machine consistently outperforms (higher AUC) the Naive Bayes classifier (significant on the 0.05 level). And thirdly, by combining information from different sources, higher prediction accuracy can be obtained.

**Table 4:** Predictive power of combined features on RB144 data set.

**AUC**	**MCC**	**SN[%]**	**SP[%]**	**Classifier**	**Patch type**	**Features**
0.77	0.30	73.8	67.1	NB	sequential	Profile+aRC
0.78	0.33	84.6	54.8	NB	topological	Profile+aRC+aBC
0.79	0.32	76.9	65.7	NB	spatial	Profile+ASA
0.79	0.34	84.3	56.2	NB	spatial	Profile+ASA+aBC+aRC

0.79	0.34	78.4	66.0	SVM	sequential	Profile+aRC
0.81	0.36	81.1	65.1	SVM	topological	Profile+aRC+aBC
0.82	0.38	81.1	66.7	SVM	spatial	Profile+ASA
0.83	0.39	82.0	66.8	SVM	spatial	Profile+ASA+aBC+aRC

Predictive power of combined features on RB144 data set using five-fold cross-validation tests. *C*-value for SVM was 1.0 and *γ*-value of RBF-kernel was 0.01. Cost factor was 5.7.

**Figure 5:**
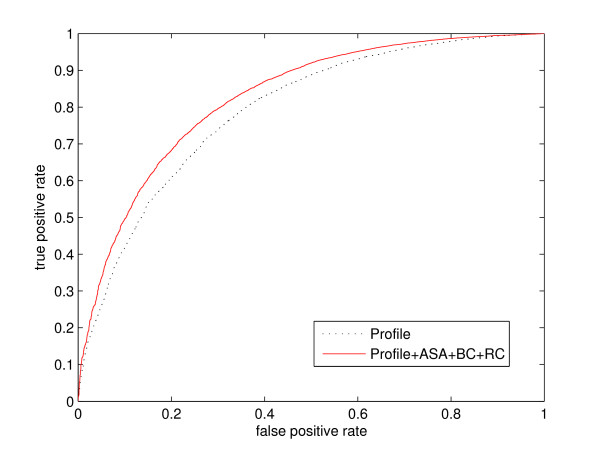
**Comparison of classifiers with different input features**. ROC curves for SVM classifiers with profile and with all input features on the RB144 data set.

The maximal AUC of 0.83 was achieved by a SVM, exploiting PSI-BLAST profiles, ASA, BC and RC as input features (Figure 4 shows an example prediction). This is a significantly (*p *< 0.05) higher accuracy than the best AUC of 0.80, accomplished by using profile information only (see last row of Table 2). Figure 5 displays the ROC curves for these two models. All other performance measures also show significant improvement: MCC increased from 0.36 to 0.39, sensitivity from 80.0% to 82.0%, and specificity raised from 65.6% to 66.8%.

There is no statistically significant difference in AUC between classifiers that utilize profile information only, and classifiers that take profiles and the retention coefficient as input - though the latter achieve marginally higher AUCs. This is explained by the fact that the profile already describes the amino acid propensity of interface residues and the additional retention coefficient, therefore, contributes little. We also evaluated the prediction performance of other classifiers such as KNN, C4.5, linear SVM and polynomial SVM but found the SVM with the RBF-Kernel to perform best (data not shown). In addition, we studied methods to balance the sample set by removal of redundant samples, down-sampling or both methods combined. But while the training time could be reduced, the resulting prediction accuracies were clearly inferior (data not shown).

### Comparison with other methods

The RB144 data set is larger and more diverse in content, and the prediction accuracies are therefore typically lower than those for smaller data sets with higher sequence similarity that are utilized in most other studies. To compare our results with previous evaluations we measured the performance of our classifier on the RB106 data set, which is almost identical to the RB109 data set used by Terribilini et al. [2,7] and Cheng et al. [24], and similar in size and sequence similarity to a data set consisting of 107 chains used by Kumar et al. [8] and other authors.

We furthermore submitted the sequences of our independent RB36 data set to the PPrint prediction server, developed by Kumar et al. [8]. An evaluation of the prediction performance of the RNABindR server [7] on the RB36 data set was omitted, since RNABindR matches a query sequence against a database of all known structures (including RB36), resulting in next to perfect predictions for known sequences.

Table 5 shows the prediction performance of our classifier with different inputs on two data sets and the results reported by other authors on similar data sets. Terribilini et al. [2,7] achieved a MCC of 0.35, utilizing a Naive Bayes classifier with amino acid frequencies as input. And Kumar et al. [8] reported a MCC of 0.28 (five-fold cross-validated), with an SVM and PSI-BLAST profiles as input on a dataset of 107 sequences.

**Table 5:** Predictor comparison with other authors.

**AUC**	**MCC**	**SN[%]**	**SP[%]**	**Classifier**	**Data set**	**Ref**.	**Features**
0.79	0.34	68	75	NB	RB106	-	Profile
0.79	0.34	68	75	NB	RB106	-	Profile+aRC
0.80	0.38	58	85	NB	RB106	-	Profile+ASA+aBC+aRC

0.81	0.36	70	76	SVM	RB106	-	Profile
0.81	0.37	70	77	SVM	RB106	-	Profile+aRC
0.84	0.43	71	81	SVM	RB106	-	Profile+ASA+aBC+aRC

0.74	0.25	52	82	SVM	RB36	-	Profile
0.75	0.27	54	83	SVM	RB36	-	Profile+aRC
0.77	0.30	52	87	SVM	RB36	-	Profile+ASA+aBC+aRC

0.67	0.34	54	95	SVM	RB36	PPrint [8]	Profile
0.86	0.50	56	93	SVM	RNAset81	[11]	Profile+IP+pA+H
-	0.28	66	74	SVM	107	[8]	Profile
-	0.35	-	-	NB	RB109	[2]	Amino Acid

Comparison with other authors for data sets similar to RB109. Sequential patch for Profile and Profile+RC, spatial patch for Profile+ASA+BC+RC.

Using profile information over a sequential patch on RB106 our SVM based classifier achieves a MCC of 0.36 (AUC = 0.81), which may be comparable with the reported MCC of 0.35 [2]. However, their value was optimized by tuning a threshold for classifying RNA binding residues. Accordingly, the specificity and sensitivity were 51% and 38%. In contrast, our simulations obtained the specificity 76% and the sensitivity 70%, which are considerably better than the above reported results.

In comparison to Kumar's result our performance estimates are clearly higher, which we attribute to differences in data sets and a comprehensive optimization of patch size and classifier parameters. When all features (Profile+ASA+aBC+aRC) are exploited and a spatial patch of size 19 is used, the prediction accuracy of our SVM based classifier increases to a MCC of 0.43 (AUC = 0.84).

The prediction results of the PPrint server [8] on the RB36 data set highlight how difficult the comparison of classifier performances is. PPrint achieves an MCC of 0.34, which is much higher than our MCC of 0.25, but both classifiers are of very similar architecture (SVM, profiles as input). The MCC however represents only a single working point on the ROC curve and the AUC (a more robust measure of prediction performance) of our classifier is considerably higher (0.74) than the AUC of 0.67 achieved by PPrint. PPrint allows the user to define a threshold to shift the working point, e.g. to balance sensitivity and specificity. We noted however that the classifier showed very high specificity despite the fact that we used the default setting (-0.2), which was reported to balance sensitivity and specificity [8]. To interpret the discrepancy in performance, it furthermore has to be taken into account that the RB36 data set is redundancy reduced against the training set of our classifier, which is not the case for the sequence set utilized by PPrint, possibly causing an advantageous bias for PPrint in this comparison.

The second highest prediction performance with an MCC of 0.5 has been reported by Spriggs et al. [11] using a SVM with an RBF kernel and only sequence features as inputs such as sequence profiles, interface propensities (IP), predicted accessibility (pA) and hydrophobicity (H). The corresponding AUC of 0.86 is even higher than the best AUC of 0.84 our classifier achieves, despite the fact that we exploit structural information in addition to sequence data and using an identical classifier architecture. The discrepancy in performance can be traced back to the data the classifier is evaluated on. The RNAset81 utilized by Spriggs et al. is smaller and only weekly redundancy reduced (up to 70% sequence similarity) while the RB106 data set used to train our classifier is larger and strongly redundancy reduced, with no more than 30% sequence similarity.

The effect of redundancy reduction (and choice of data set) on prediction accuracy is documented by Cheng et al. [24]. They compared the performance of their classifier on three data sets (RBP86, RBP109, RBP107) with different degrees of redundancy reduction, confirming that the prediction accuracy increases with the degree of sequence similarity.

Recently, Cheng et al. [24] introduced smoothed sequence profiles to take the dependency between neighboring residues into account and reported large improvement. Smoothed profiles adopted a simple approach to obtain their values by averaging the normal profile values in a certain window. We applied this approach to our SVM and Naïve Bayes classifiers. However, in both cases, we did not observe improvements with respect to prediction accuracy.

The highest prediction performance of our classifier with a MCC of 0.30 and an AUC of 0.77 on the RB36 data set was achieved, using a SVM and all features (Profile+ASA+aBC+aRC). It is however to note that the improvement in prediction accuracy by adding topological or structural features to the purely sequence based profile information is comparatively small (AUC increases from 0.74 to 0.77).

## Conclusion

Residues that participate in RNA-protein interfaces show different characteristics, which can be derived from sequence, structure, graph-topology, and physicochemical properties. Previous work studied different residue properties in protein-RNA interfaces, such as the amino acid doublet propensity [6], electrostatics, conservation and surface cleft arrangement [12], as well as atomic packing patterns [25]. In addition, many authors used machine learning methods to predict protein RNA-binding sites directly from sequences and their performance has been strictly examined on large data sets with cross-validation or on independent data sets. Aggregation of these types of features with already developed sequence-based methods will gain higher prediction performance.

In this study, we particularly examined the graph-theoretic properties of residue contact maps derived from protein structures and found a number of features, such as betweenness centrality and status, which show higher or compatible predictive power to already known structural features such as the ASA. Taking them into account, sequence-based methods can improve prediction accuracy, and the highest AUC of 0.83 (MCC = 0.39) was achieved by a Support Vector Machine with an RBF-Kernel, using a spatial patch of size 19, and profiles, accessible surface area, betweenness-centrality and retention coefficient as input. The *blind test*, deemed as the most strict test, using newly solved protein-RNA complexes to test the prediction performance gives an AUC of 0.77 (MCC = 0.30) on the RB36 data set.

We also compared the prediction performance of Naive Bayes classifiers and Support Vector Machines and found that the latter generally improve accuracies (AUC) by about 5%. However, the Naïve Bayes method requires less computing time and therefore remains an attractive choice for large scale data analysis. We implemented a web application (PRIP) to predict binding residues in Protein-RNA interfaces from sequence information . The data sets we generated and utilized for our experiments can be downloaded from the same web page.

RNA-protein recognition is known to be a surprisingly complicated and diverse process, which has been investigated by different experimental techniques and theoretical methods. Those studies provide a variety of information on different aspects, useful to build up a systemic description of this important protein function. In this study, one one hand, we exploit different resources to improve the prediction accuracy. On the other hand, we employ network theory to describe the collective properties of interface residues, which may more accurately reflect the nature of binding process as different interactions in the interfaces are largely responsible for binding affinity and specificity. Finding more accurate interpretation of those interactions is crucial for this problem, which forms our future research.

## Methods

### Data sets

We downloaded the RB147 and the RB109 data sets created by Terribilini et al. [2,7] from the RNABindR web server at . RB147 is a redundancy reduced set (sequence similarity smaller than 30%), composed of 147 RNA-binding protein chains extracted from the PDB. A distance cutoff of 5 Å was used to identify binding residues. Note that while RB147 is the largest data set currently available it is still significantly biased toward ribosomal proteins (77) and aminoacyl-tRNA synthetases (19). All chains shorter than 40 residues were removed to allow the calculation of spatial and topological patches of reasonable sizes. The new data set RB144, consists of 144 proteins, all solved before 2006, with 4304 binding and 27932 non-binding residues.

RB109 is a smaller set of 109 RNA-binding protein chains extracted from the PDB in 2004, which we used for comparison with other authors. ENTANGLE [26] was used to identify interface residues. The data set is redundancy reduced, with a sequence similarity threshold of 30%. There is an overlap of 66 chains between RB109 and RB147. For some chains of the RB109 data set short segments of the sequences in RB109 were lacking corresponding structural information in the related PDB file. In these cases, the sequence contained in the PDB file was utilized. Similarly to RB144, chains shorter than 40 residues were removed, resulting in a new data set RB106, with 106 chains, and 3543 binding and 20264 non-binding residues. Finally, we created a new, independent test set RB36 by extracting all structures of Protein-RNA complexes from the PDB that were added after January 2006. We filtered for structures with a resolution better than 3.5 Å and removed all chains shorter than 40 residues. We then performed a redundancy reduction to ensure that none of the chains showed a sequence similarity of more than 30% within the data set and to the RB144 data set. A distance cutoff of 5 Å was used to annotate interface residues.

### Training and testing

For training and testing a cross-validation and feature evaluation framework in Java (1.6.0) was implemented that utilized the Naive Bayes and the SVM classes of the WEKA [27] library to perform predictions. A logistic model was fitted to the output of the SVM (WEKA option "-M") to estimate classification probabilities required to calculate the AUC. Since the data sets are heavily unbalanced, training samples were weighted in accordance to the overall ratio of positive and negative samples (cost factor 5.7). Cross-validation sets were generated by splitting the data sets into sub sets with roughly equal numbers of chains.

### Significance and performance tests

To assess the discriminative power of a predictor the *Area under the ROC curve *(AUC) [28], Matthews Correlation Coefficient (MCC) [29], Sensitivity and Specificity were calculated. MCC, Sensitivity (SN) and Specificity (SP) are defined as follows [29]:

(1)

(2)

(3)

where *tp *is the number of true positives, *fp *is the number of false positives, *tn *is the number of true negatives and *fn *is the number of false negatives. An MCC of +1 indicates perfect correlation between the observed and the predicted classes of the samples, a MCC of -1 perfect anti-correlation, and a MCC of zero no correlation at all.

The Receiver Operating Characteristic (ROC) curve plots the true positive rate over the false positive rate [30]. MCC, SN and SP represent a specific point on the ROC curve and are only reported for comparison with other work. In contrast, the AUC is a robust performance measure that is invariant to the prior probabilities of class membership and does not depend on a specific working point [28]. The AUC ranges from 0.5 (equivalent to random choice) to 1.0 (perfect classification).

The variability in the calculated performance measures over cross-validation runs were estimated by the 95% confidence intervals, which are calculated as:

(4)

where *σ*_*m *_is the standard deviation of the performance measure and *n *is the number of folds times the number of repeats of the cross-validation experiment.

To assess the significance of differences in prediction accuracy (AUC) between models, **paired **two-tailed *t*-tests [31] were performed:

(5)

where *n *is the number of runs. *x*_*i *_and *y*_*i *_are prediction accuracies (AUCs) of the two models *x *and *y *for the *i*-th input sample, and  and  are the means of *x*_*i *_and *y*_*i *_over all input samples. From the *t*-value a *p*-value can be derived, and *p *< 0.05 indicated statistically significant differences in the prediction performance.

### Features

We compared various features with respect to their predictive power for interface residues in RNA-protein binding sites. In some cases averaged feature values (*r*) for residues were calculated as the mean value over the individual feature values *f*(*r*_*i*_) for the residues of a patch:

(6)

with *f*(*r*_*i*_) being the feature value for the *i*-th residue within a patch of size *n*. In the following we describe only features for individual residues, if not stated otherwise. Patch bound versions of features were calculated as described above.

Most topological features of contact graphs described in this paper were computed utilizing the JUNG library . Contact graphs were derived from the spatial coordinates of protein structures with a cutoff distance of 8 Å between the *C*_*α*_-atoms of the residues. We also evaluated other methods to generate contact graphs based on the distance of side-chain or *C*_*β*_-atoms, but found no significant differences in the resulting prediction performances.

Sequence profiles encode evolutionary information about amino acids and have successfully been employed for binding site prediction before [8]. A sequence profile is described by *n *vectors of length 20 that represent the log-likelihood for different amino acids in a specific sequence position, with *n *being the length of the sequence. Profiles were generated by PSI-BLAST [9] with three iterations and an expectation value of 0.001.

Amino acid indices express the propensity of amino acids for specific physicochemical or structural environments. They range from simply hydrophobicity indices to scales derived from tertiary protein information. We downloaded 544 amino acid propensity scales from Release 9.1 of the AAIndex database [17] and evaluated their predictive power for RNA-protein binding sites (see Section Methods). The **Accessible surface area **(ASA) was calculated using the DSSP [32] software. It represents the residue water exposed surface in Å^2^. The **relative Accessible surface area **(rASA) is the ASA, normalized by the values derived from the tripeptide extended conformation of the chain.

**Density **describes the spatial compactness of a patch as the averaged euclidean distance *d*_*e*_(·,·) between the *C*_*α*_-atom of the center residue *r*_*c *_and the *C*_*α*_-atoms of all other patch residues *r*_*i*_. For a patch of size *n *the Density *D*(*r*_*c*_) is computed as

(7)

The **Degree ***G*(*v*) of a vertex in a topological patch is the number of edges that connect the vertex with its neighbor vertices.

**Betweenness centrality **measures how frequently a vertex is on the shortest path between all other vertex pairs within the contact graph of a protein chain of length *n*. Since the chains vary in length the normalized Betweenness centrality *BC*(·) for a vertex *v *is calculated, which is defined as

(8)

with *V *is the set of vertices, *σ*_*st *_is the number of shortest paths from *s *to *t*, and *σ*_*st*_(*v*) is the number of shortest paths from *s *to *t *that pass through vertex *v*.

The **Status ***S*(·) is the sum over all geodesic distances *d*_*g*_(·,·) between the vertex of interest *v *and all other vertices *v*_*i *_within the contact graph of a protein chain with *n *residues:

(9)

The **Eccentricity ***E*(·) is the greatest geodesic distances *d*_*g*_(·,·) between the vertex of interest *v *and any other vertex *v*_*i *_within the contact graph of a protein chain:

(10)

**Closeness ***C*(·) is calculated as the mean geodesic distance *d*_*g*_(·,·) between the vertex of interest *v *and any other vertex *v*_*i *_within the contact graph of a protein chain of length *n*:

(11)

The **Cluster coefficient ***CC*(·) for a vertex *v *is the proportion of edges between its direct neighbors divided by the number of all possible edges between them. It is calculated as

(12)

with *k *is the number of neighbors of vertex *v *and *n*_*e *_is the number of edges between the neighbors.

## Authors' contributions

SM conceived the method, implemented the software and authored the first draft. ZY participated in the design of the method and data set preparation and helped to draft the manuscript. All authors read and approved the final manuscript.
